# Supplementary material for: Dynamic imaging of lithium in solid-state batteries by operando electron energy-loss spectroscopy with sparse coding
Source: Nat Commun. 2020 Jun 4;11:2824. doi: 10.1038/s41467-020-16622-w (PMC7272654; doi:10.1038/s41467-020-16622-w)
Supplement: Supplementary file 1 — Supplementary Information [file 41467_2020_16622_MOESM1_ESM.pdf]

Supplementary Information

Dynamic imaging of lithium in solid-state batteries by *operando* electron energy-loss spectroscopy with sparse coding

Yuki Nomura et al.

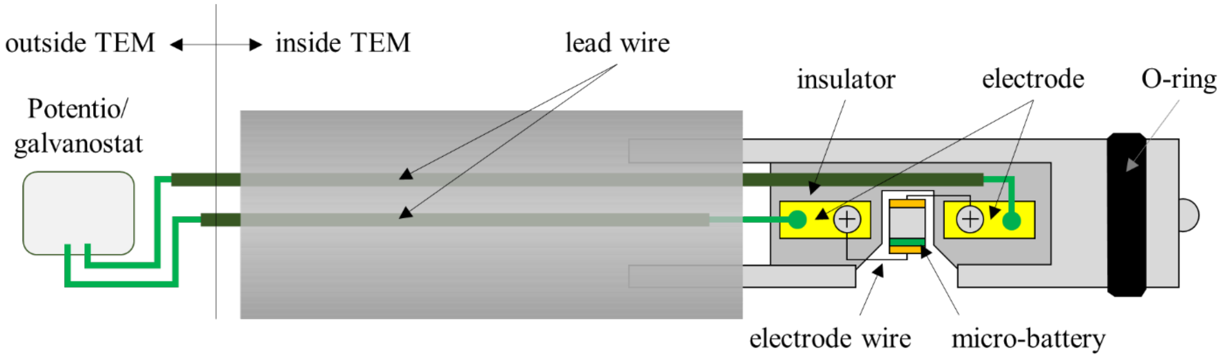

Supplementary Figure 1. Configuration of the biasing TEM holder and the method to bias the micro-battery. The electrode wires and micro-battery were connected using silver paste. Only the cathode side of the micro-battery was thinned by focused-ion beams.

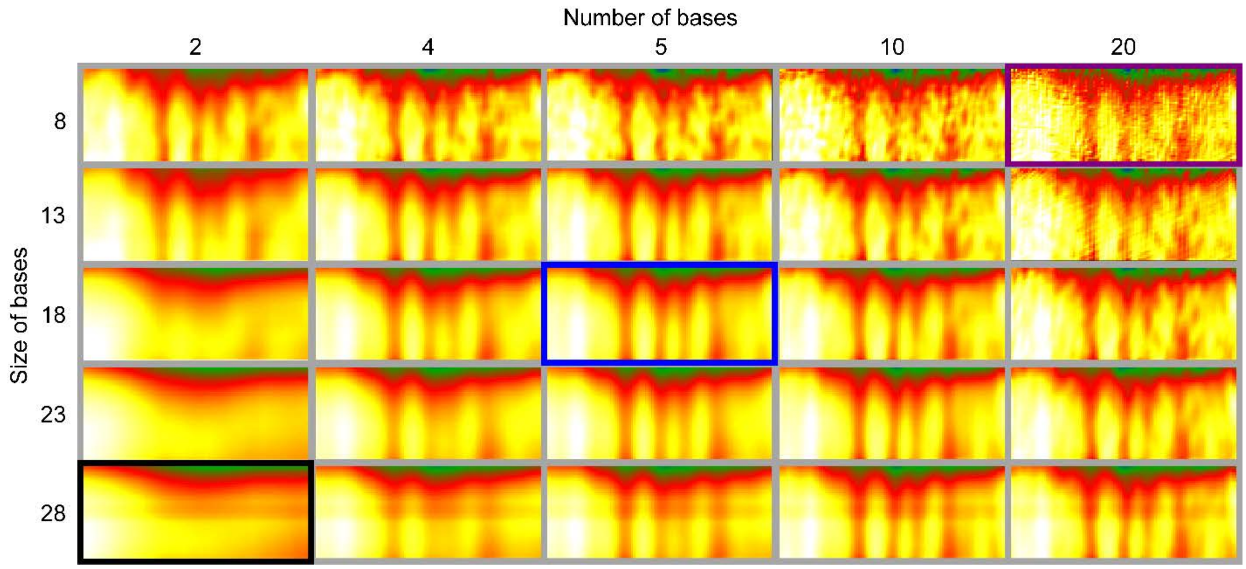

Supplementary Figure 2. Comparison of images processed with different hyper-parameters (size and number of bases) in sparse coding. The optimized hyper-parameters are 18 pixels and 5 bases (blue rectangle).

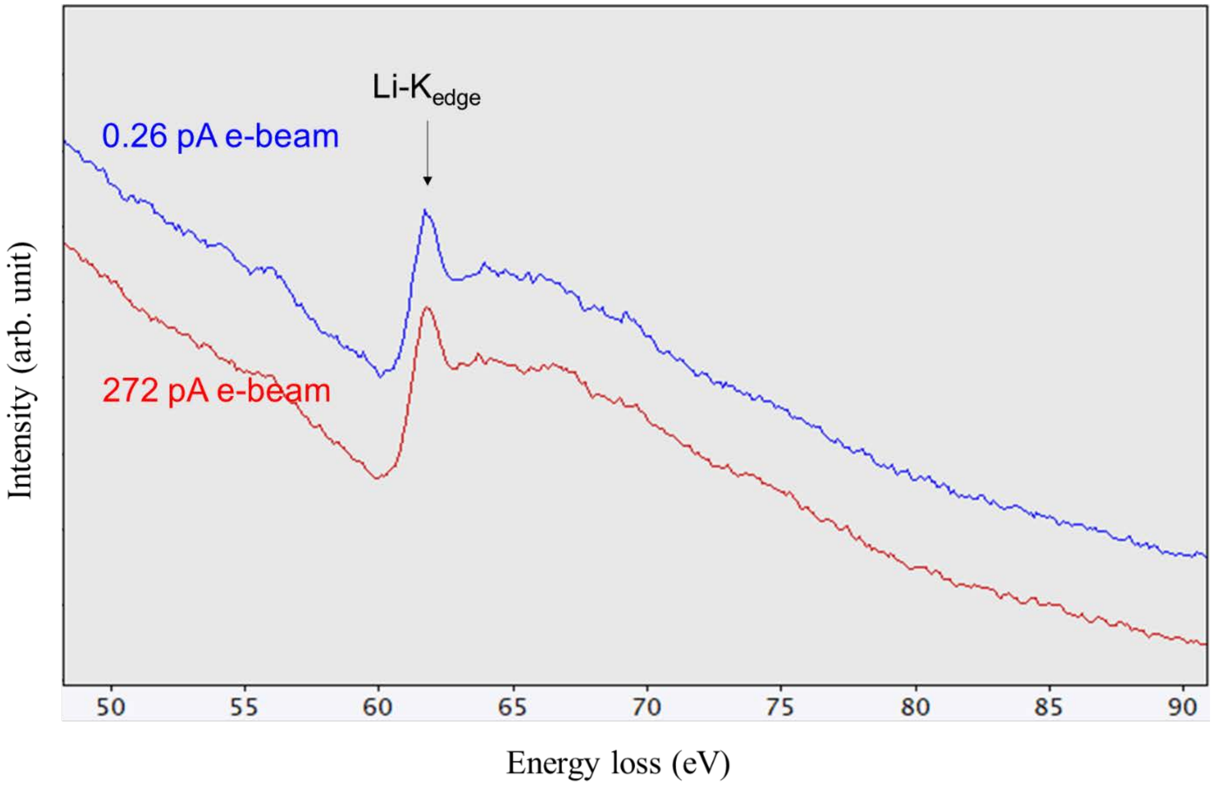

Supplementary Figure 3. Comparison of two Li-K EEL spectra acquired using 0.26 and 272 pA e-beams. The STEM probes were positioned at the same $LiCoO_2$ region with the same probe size (about 5 nm).

Supplementary Note 1. Sparse coding

Sparse coding techniques have been applied to electron microscopy images[1,2]. The most important difference in our method is optimization of the hyper-parameters. In previous studies, the reliability of the reconstructed images was not guaranteed because the hyper-parameters (size of the bases, number of bases, and sparsity) were not optimized. We optimized the hyper-parameters using training images (Fig. 3a–c, high quality Li maps) and the cross-validation method, which estimates the hyper-parameters to provide the minimum error. The quality of the reconstructed image significantly depended on the hyper-parameters. A comparison of images processed with different hyper-parameters in sparse coding is shown in Supplementary Fig. 2. Figure 3d was used as the test image, and Fig. 3b and c were used as the training images. When smaller size and larger number of bases were used as the hyper-parameters (e.g., size of 8 pixels, number of bases of 20, top-right purple rectangle), the noise could not be removed. Conversely, when larger size and smaller number of bases were used (e.g., size of 28 pixels, number of bases of 2, bottom-left black rectangle), the noise was successfully removed, but the contrast of the $LiCoO_2$ domain disappeared. Using the optimized hyper-parameter (size of 18 pixels, number of bases of 5, centre blue rectangle), efficient noise reduction and preservation of the $LiCoO_2$ domain contrast were simultaneously achieved. The results showed that hyper-parameter tuning using the training images is important for image processing with sparse coding.

Supplementary Note 2. Electron beam effect

We consider that the electron beam effect can be categorized into two types for observing and evaluating battery materials using TEM. The first type is deterioration of the crystal structure by atom displacement, e-beam sputtering, e-beam heating, electrostatic charging, and radiolysis[3]. In $LiCoO_2$, it is well known that crystal deterioration first appears as “cation mixing” between Li and Co sites. The relationship between the electron dose and cation mixing has been reported by Shim *et al*.[4]. From their STEM imaging conditions, the calculated threshold dose for cation mixing in $LiCoO_2$ was more than 2.2×10^7 [electron/Å^2]. However, the total dose in our study was 4.3×10^5 [electron/Å^2], which is two orders of magnitude lower than the threshold value. Thus, we believe that deterioration of the crystal structure hardly occurred in our experiment. The second type is Li-ion diffusion induced by electric charging of the TEM sample. For example, if the illuminated area is positively charged, Li ions with positive charge might move away from the area. To clarify this effect, we compared two Li-K EEL spectra acquired using weak and strong e-beams (different dose rates), where we assumed that the extent of the electric charging depended on the dose rate. If the second type is effective, the Li-K intensity changes depending on the dose rate. Blue spectrum in Supplementary Fig. 3 was acquired using a 0.26 pA probe with 100 s exposure. Red spectrum was acquired using a 272 pA probe with 0.1 s exposure, which is almost same dose rate as our *operando* STEM-EELS. For both of the spectra, the STEM probes were positioned at the same $LiCoO_2$ region with the same probe size (about 5 nm). The results showed that the Li-K intensities were almost the same. Thus, we concluded that electric charging did not occur and affect Li-ion movement under our STEM-EELS conditions because of the high electron conductivity of $LiCoO_2$.

Supplementary Methods. Biasing TEM holder

The configuration of the biasing TEM holder is reported in our previous study[5]. A schematic of the micro-battery on our biasing TEM holder is shown in Supplementary Fig. 1. The sample preparation procedure was as follows. First, Cu electrode wires were connected to both sides of the micro-battery using silver paste. Only the cathode side of the battery was then thinned using 30 and 8 kV focused Ga-ion beams. Finally, the thinned TEM sample with the Cu electrode wires was placed on the two biasing electrodes of the TEM holder. The charge and discharge curves in Fig. 1b were measured using this system in the transmission electron microscope.

Supplementary References

1. Stevens, A., Yang, H., Carin, L., Arslan, I. & Browning, N. D. The potential for Bayesian compressive sensing to significantly reduce electron dose in high-resolution STEM images. *Microscopy* **63**, 41–51 (2014).

2. Stevens, A. *et al.* A sub-sampled approach to extremely low-dose STEM. *Appl. Phys. Lett.* **112**, 043104 (2018).

3. Egerton, R. F., Li, P. & Malac, M. Radiation damage in the TEM and SEM. *Micron* **35**, 399–409 (2004).

4. Shim, J. H., Kang, H., Kim, Y. M. & Lee, S. In situ observation of the effect of accelerating voltage on electron beam damage of layered cathode materials for lithium-ion batteries. *ACS Appl. Mater. Interfaces* **11**, 44293–44299 (2019).

5. Hirayama, T. *et al.* Advanced electron holography techniques for in situ observation of solid-state lithium ion conductors. *Ultramicroscopy* **173**, 64–70 (2017).
