# Supplementary material for: Dynamic imaging of lithium in solid-state batteries by operando electron energy-loss spectroscopy with sparse coding
Source: Nat Commun. 2020 Jun 4;11:2824. doi: 10.1038/s41467-020-16622-w (PMC7272654; doi:10.1038/s41467-020-16622-w)
Supplement: Supplementary file 3 — Description of Additional Supplementary Files [file 41467_2020_16622_MOESM3_ESM.pdf]

Description of Additional Supplementary Files

Supplementary Movie 1:

Movie of Li-ion dynamics in a solid-state battery.
